# Extracellular payload release from non-internalizing antibody–drug conjugates: mechanisms and linker technologies

**DOI:** 10.1080/10717544.2026.2645769

**Published:** 2026-03-22

**Authors:** Chenxi Feng, Rui Lou, Shipeng Chen, Furong Lin, Jiaqi Ge, Yuwen Zhang, Chaolong Lin, Chenghao Huang

**Affiliations:** a State Key Laboratory of Vaccines for Infectious Diseases, Xiang An Biomedicine Laboratory, Department of Laboratory Medicine, School of Public Health, Xiamen University, Xiamen, People's Republic of China; b National Institute of Diagnostics and Vaccine Development in Infectious Diseases, NMPA Key Laboratory for Research and Evaluation of Infectious Disease Diagnostic Technology, Xiamen University, Xiamen, People's Republic of China; c Department of Laboratory Medicine, Xiamen Key Laboratory of Genetic Testing, The First Affiliated Hospital of Xiamen University, School of Public Health, Xiamen University, Xiamen, People's Republic of China

**Keywords:** Antibody–drug conjugates, noninternalizing, payload release, linker technologies, bystander effect

## Abstract

Antibody‒drug conjugates (ADCs) have taken on a significant role in precision oncology. These molecules, referred to as 'biological missiles', can deliver cytotoxic drugs directly to cancer cells. Traditional ADCs rely on endocytosis and intracellular release of payloads, but this approach becomes complicated due to issues like antigen loss, tumor heterogeneity, and impaired endocytosis, leading to therapeutic resistance. To address these challenges, noninternalizing ADCs have been developed, utilizing extracellular payload release methods. These structures employ advanced linker technologies to ensure stability in vivo and selective activation in the tumor microenvironment, achieving effective cytotoxic diffusion among tumor cells through the 'bystander effect'. This review discusses the evolution from early linker designs to complex methods based on tumor-specific conditions or external triggers. It also examines the categories of noninternalizing ADC linkers and the latest developments in clinical research, exploring prospects for enhancing the efficacy and safety of ADCs in oncology applications.

## 1. Introduction

**Figure 1.**
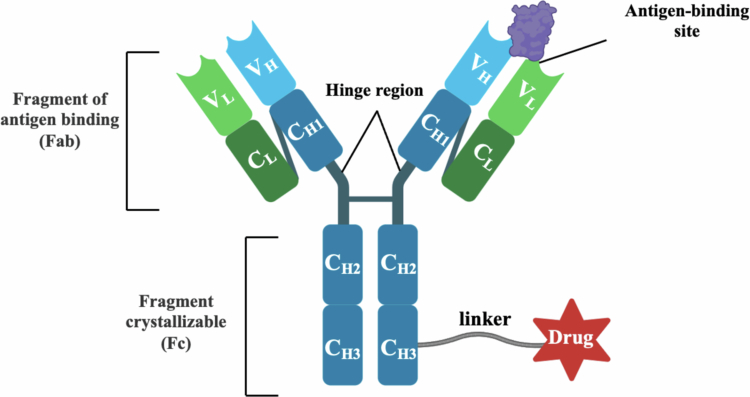
Structure of antibody–drug conjugate.

The conceptual foundations of targeted cancer therapy date to the early twentieth century, when Paul Ehrlich articulated the ‘magic bullet’ hypothesis, envisioning agents capable of selectively eradicating diseased cells while sparing healthy tissues. Subsequent advances in monoclonal antibody engineering and linker chemistry culminated in the development of antibody–drug conjugates (ADCs), which couple tumor-selective recognition with highly potent cytotoxic payloads to expand the therapeutic window (Strebhardt and Ullrich 2008). An ADC typically comprises three fundamental components – a targeting antibody, a cytotoxic warhead and a chemical linker – and has emerged as a major class of precision oncology therapeutics, often described as ‘biological missiles’ (Wang et al. 2023) (Figure 1). Traditionally, ADC efficacy is reliant on the internalization mediated by target binding and subsequent intracellular release of cytotoxic payloads (Sahoo et al. 2022). Nevertheless, this internalization-centric approach faces substantial clinical obstacles such as diminished expression (Larose et al. 2025), loss (Chen et al. 2016), or mutation of tumor antigens, coupled with tumor heterogeneity, which are major contributors to ADC resistance (Jiang et al. 2024). The therapeutic action of ADCs involves binding to antigens on the cell surface, followed by endocytosis and payload release in the lysosome – critical steps for inducing cytotoxicity (Zhang et al. 2021). Any impairment in endocytosis or endosomal trafficking disrupts ADC effectiveness (Zhitomirsky and Assaraf 2016). Besides, factors like elevated lysosomal pH (Macintyre and Cutler 2006) and lysosomal sequestration (Lou et al. 2006; Zhitomirsky and Assaraf 2016; Hussein et al. 2021) are known to induce resistance to ADCs (Chen et al. 2022; Li et al. 2025). Additionally, poorly internalizing or noninternalizing targets pose significant challenges to standard ADC designs.

In response to these challenges, the concept of noninternalizing ADCs has been introduced (Balamkundu and Liu 2023). Importantly, the term ‘noninternalizing’ is operational rather than absolute. Many such targets display low-level or delayed uptake, such that therapeutic activity may derive from a composite of limited intracellular release from a minor internalized fraction together with dominant extracellular payload liberation (Ashman et al. 2022). These constructs depend on linkers adept at extracellular payload release. The efficacy and safety of noninternalizing ADCs hinge on the linker's ability to remain highly stable in systemic circulation, thereby minimizing systemic toxicity, and to be selectively activated within the tumor microenvironment (Santin et al. 2025), releasing active agents that exert a ‘bystander effect’ to eradicate adjacent tumor cells (Jerjes et al. 2020).

We operationally define a ‘slowly internalizing’ target as one that sustains surface antigen accessibility over a therapeutically relevant time window, rather than one that fails to internalize altogether. CD20 serves as a prototypical example: 16 hours after binding with type II anti-CD20 antibodies, approximately 70–75% of surface CD20 remained accessible, whereas type I antibodies reduced surface CD20 to about 15–25% despite comparable baseline binding, indicating binding modality-dependent differences in internalization kinetics (Shih et al. 1994). Time-course immunofluorescence and radiolabeled tracing further demonstrated that the anti-CD20 antibody (1F5) exhibited minimal internalization at early time points (1–4 hours), with intracellular accumulation only occurring after prolonged incubation (16–18 hours) (Mattes 2002). Applying this definition, CEACAM5 should likewise be classified as slowly internalizing: although often described as noninternalizing, electron microscopy, radiotracing, and flow cytometry studies have shown gradual internalization of anti-CEACAM5 antibodies over hours to days, this is consistent with a membrane turnover-mediated mechanism(Liao-Chan et al. 2015; Shih et al. 1994). PD-L1 further supports this framework: radiolabeled antibody assays revealed that only approximately 25% of bound antibody was internalized after 24 hours, with about 75% remaining membrane-associated, leading to its explicit characterization as ‘slowly internalizing’ (Heskamp et al. 2015).

Recent studies have found that noninternalizing ADCs offer an alternative mechanism that does not strictly rely on cellular endocytosis. These ADCs, after binding to their target antigen, do not immediately internalize (Parakh et al. 2021). Instead, they utilize the characteristics of the tumor microenvironment (TME), such as a lower pH, high levels of extracellular proteases like cathepsin B (Yap et al. 2019), or reducing substances like glutathione, to specifically cleave the linker extracellularly and release small molecule cytotoxic drugs (Bargh et al. 2019; Li et al. 2021). This mechanism significantly expands the potential target range of ADCs, making tumor-associated antigens or extracellular matrix components (Hooper et al. 2022) that are noninternalizing or have low internalization efficiency viable targets (Li et al. 2021). It provides new insights for overcoming many challenges in solid tumor therapy such as heterogeneity, antigen barriers, and resistance. This review comprehensively examines the design rationale, mechanistic categories, and recent progress in noninternalizing ADC linker development, offering a perspective on future directions to facilitate technological innovation in this domain.

## 2. Mechanisms of internalizing and noninternalizing ADCs

### 2.1. Classic pathway of traditional internalizing ADCs

Traditional ADCs mediate targeted cytotoxicity through antigen-dependent internalization (Jin et al. 2022; Mazahreh et al. 2023). Upon binding to cell surface antigens, the ADC-antigen complex is internalized via endocytosis, trafficked to endosomes, and ultimately delivered to lysosomes (Lai et al. 2022). Within the acidic lysosomal milieu, the linker is cleaved – either by low pH, proteases, or high glutathione concentration – releasing the potent cytotoxic payload (Zhong and D'Antona 2021). This payload then induces cell death by disrupting critical processes such as microtubule function or DNA integrity (Hafeez et al. 2020). ADCs comprise three critical components: a monoclonal antibody, a linker, and a cytotoxic payload. The payload is connected to the monoclonal antibody through the linker. The monoclonal antibody is designed to recognize and bind to specific antigens expressed on the surface of cancer cells, serving as a precise delivery vehicle that localizes the cytotoxic payload to tumor tissue (Sasso et al. 2023). Upon successful delivery, the payload exerts its potent antitumor activity, leading to the targeted destruction of cancer cells (Xu 2015).

**Figure 2.**
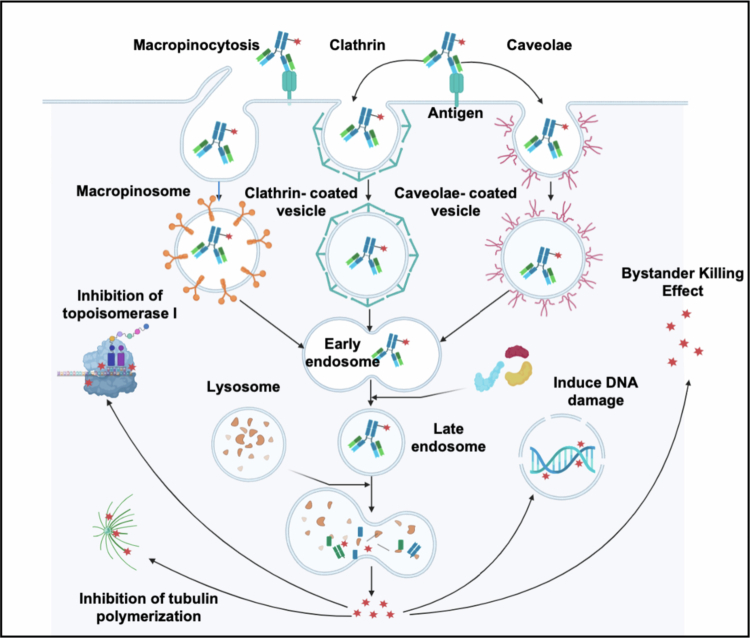
The mechanism of internalizing ADCs. Conventional ADCs are internalized through macropinocytic or clathrin- and caveolae-mediated endocytic pathways and subsequently trafficked to lysosomes, where proteolytic processing of the linker enables intracellular liberation of the cytotoxic payload.

As of the end of 2024, the U.S. Food and Drug Administration (FDA) had approved 15 ADCs for clinical use, with over 400 ADCs actively conducting clinical trials (Hong et al. 2023). Currently, the indications for ADC drugs primarily focus on hematological malignancies and some solid tumors (Wittwer et al. 2023). They are typically administered intravenously and transported to tumor sites via the blood and lymphatic systems. The ADC-antigen complex enters the cell via clathrin- or caveolin-mediated endocytosis and is encapsulated in endosomes (Banushi et al. 2023; Barman and Drolia 2024). Early endosomes mature into late endosomes before fusing with lysosomes. ADCs with cleavable linkers undergo cleavage – such as hydrolytic, proteolytic, or reductive cleavage within early or late endosomal compartments (Erickson et al. 2006; Dai et al. 2024). After endosome‒lysosome fusion, the ADCs are completely hydrolyzed by cathepsin B, plasmin, etc, (Sievers and Senter 2013). This process releases the active payload into the cytosol, where it induces apoptosis by mechanisms including DNA insertion and microtubule synthesis inhibition (Birrer et al. 2019). Upon apoptosis of the target cells, the liberated payload diffuses freely, exerting cytotoxic effects on neighboring tumor cells – a phenomenon known as the bystander effect (Figure 2) (Staudacher and Brown 2017).

### 2.2. The new paradigm of extracellular activation in non-internalizing ADCs

Noninternalizing ADCs constitute a paradigm shift in targeted cancer therapy by exerting cytotoxic effects independent of antigen‒antibody complex endocytosis (Joubert et al. 2017). Their efficacy relies on the extracellular cleavage of the linker within the tumor microenvironment (TME), releasing membrane-permeable payloads that diffuse into cells. This strategy significantly expands the targetable antigen repertoire to include poorly internalizing antigens (e.g. CEACAM5, CD20) (DiJoseph et al. 2006; Zhu et al. 2023). It enhances solid tumor penetration by facilitating deeper diffusion of small-molecule drugs, overcoming the ‘antigen barrier’ and promoting a potent bystander effect against heterogeneous tumors. Furthermore, this approach potentially circumvents resistance mechanisms associated with impaired internalization or drug efflux pumps.

Recent research indicates that lysed tumor cells release high concentrations of cellular contents, such as glutathione and proteases, into the tumor microenvironment (Vidyasagar et al. 2010). This observation suggests that for ADCs with cleavable linkers, the separation of antibody and payload may occur extracellularly, independent of the endocytic pathway. Leveraging this principle, researchers have developed noninternalizing ADCs. These ADCs are designed such that, upon target binding by the monoclonal antibody, linker cleavage occurs within the specialized biochemical conditions of the tumor microenvironment, resulting in the extracellular release of the active payload, which can directly kill tumor cells (Fu et al. 2022).

Noninternalizing ADCs offer distinct advantages over their internalizing counterparts. First, noninternalizing ADCs do not rely on receptor-mediated endocytosis, enabling them to target a broader range of noninternalizing antigens present in the tumor microenvironment, such as characteristic metabolites, stromal cells, and vascular fibrous tissues. This capacity makes them particularly suitable for tumors with high receptor heterogeneity or low expression levels of conventional internalization receptors. Second, given the complex and variable metabolic landscape within tumors, internalizing ADCs can suffer from the degradation of payloads during intracellular transport and lysosomal processing, potentially diminishing their efficacy – a challenge noninternalizing ADCs circumvent.

Moreover, the large molecular size of antibodies in ADCs limits tissue penetration. Peripheral tumor cells often express similar specific antigens, creating an ‘antigen barrier’ (El Emir et al. 2007). In dense tumor tissues, internalizing ADCs predominantly bind peripheral tumor and stromal cells, impeding access to deeper tumor cells. In contrast, noninternalizing ADCs release their payloads extracellularly, leveraging small molecules with superior penetration capabilities that reach deeper tumor regions and maximize the bystander effect (Dennis et al. 2007). Additionally, certain tumor cells evade internalizing ADCs by downregulating or mutating surface receptors and increasing drug efflux, contributing to resistance (Loganzo et al. 2015; Barok et al. 2014). Noninternalizing ADCs, targeting stromal receptors while bypassing cellular efflux pathways, effectively reduce the incidence of resistance.

**Figure 3.**
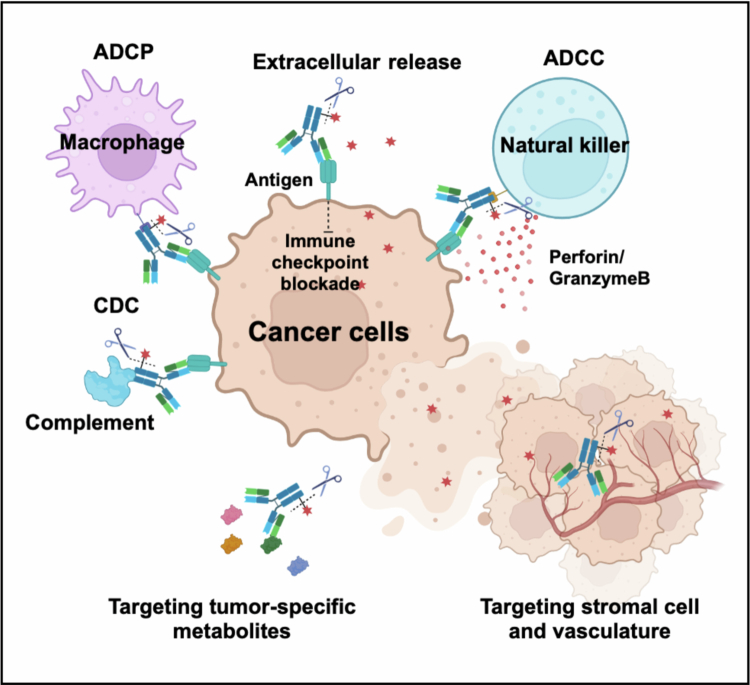
The mechanism of noninternalizing ADCs. Non-internalizing ADCs exert antitumour activity through extracellular payload release, enabling targeting of stromal components, while surface-retained antibodies engage CDC, ADCC and ADCP to generate complementary antitumour effects.

Importantly, studies show that antibodies targeting noninternalizing sites accumulate in lipid rafts – rich in glycosphingolipids and cholesterol – within the plasma membrane. These domains, known as lipid rafts, sequester molecules from internalization, allowing sustained surface display, signal transduction affecting cell growth and function, and mediating ADCC, ADCP, and CDC effects (Polyak et al. 1998; Harder and Engelhardt 2004). These advantages highlight the unique potential of noninternalizing ADCs, setting them apart from traditional internalizing strategies (Figure 3).

## 3. Key technologies of noninternalizing ADCs: linker chemistry

**Figure 4.**
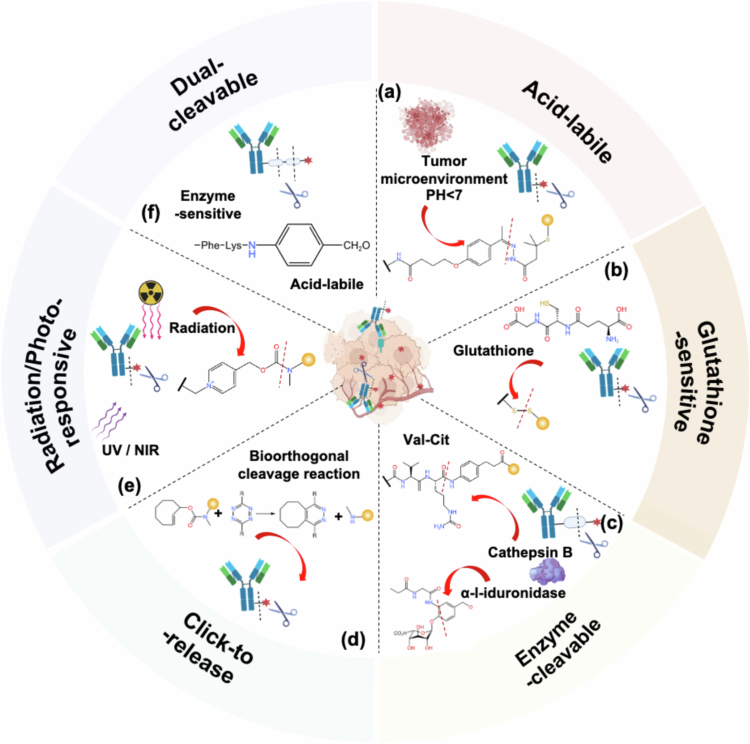
Progress in the design of extracellularly cleavable ADC linkers. (a) Acid-labile linker: Specifically cleaved in low pH environments to achieve targeted release. (b) Glutathione-sensitive linker: Utilizes elevated intracellular glutathione levels in tumor cells to trigger drug release. (c) Enzyme-cleavable linker: cleaved by specific enzymes (e.g. Cathepsin B, *α*-l-iduronidase) for bioresponsive drug release. (d) Click-to-release linker: Enables controlled, rapid release via a reverse click chemistry reaction. (e) Radiation/Photo-responsive linker: Cleaved upon light or radiation exposure for spatiotemporal release control. (f) Dual-cleavable linker: Incorporates two distinct cleavage mechanisms to enhance release selectivity and efficiency.

Linkers utilized in ADCs are generally categorized into cleavable and noncleavable types. ADCs with noncleavable linkers predominantly depend on the ‘endocytosis–lysosomal degradation–release’ pathway to liberate their therapeutic payload. Conversely, noninternalizing ADCs, which do not penetrate cell interiors, largely employ cleavable linkers (Figure 4). An appropriate linker ensures the stability of the ADC during systemic circulation, preventing premature payload detachment and off-target toxicity. Upon reaching the tumor microenvironment, the linker must ensure rapid cleavage for timely payload release.

Cleavable linkers are further subdivided based on their cleavage mechanisms, including acid-labile linkers, enzyme-sensitive linkers, photo-responsive linkers, click-to-release linkers, glutathione-sensitive linkers, and metal-mediated cleavable linkers, etc. This manuscript will provide an in-depth exploration of the cleavage mechanisms associated with various cleavable linkers, alongside discussing the latest advancements in this field.

### 3.1. Cleavage triggered by the internal environment

#### 3.1.1. Acid-labile linkers

The design strategy for acid-labile linkers capitalizes on the pH differential between lysosomes (pH 4.5–5.0) and normal tissues to achieve targeted drug release within tumor environments. The team led by Nitin K. Damle demonstrated that an acid-sensitive AcBut linker conjugating Rituximab with CalichDMH significantly inhibited the progression of subcutaneous non-Hodgkin lymphoma BCL xenografts (DiJoseph et al. 2006). In contrast, rituximab conjugated to CalichDM via an acid-stable amide linker showed inferior efficacy in the same model. Remarkably, the authors also found that an acid-sensitive ADC targeting the noninternalizing antigen CD20 showed enhanced therapeutic effectiveness.

Since the antibody is not internalized after binding to CD20, ADCs with acid-stable linkers fail to deliver calicheamicin intracellularly to elicit cytotoxic effects, leading to suboptimal efficacy. However, tumor cells exhibit distinct metabolic properties, such as active glycolysis, causing extracellular acidification (Justus et al. 2013). This acidified microenvironment around BCLs may facilitate the hydrolysis of the amide bonds in acid-unstable linkers, enabling the extracellular release of CalichDMH to target BCLs. Consequently, acid-sensitive linkers like AcBut broaden the spectrum of antigens suitable for ADC targeting, allowing even noninternalizing antigens to be leveraged in anticancer therapies.

Carcinoembryonic antigen-related cell adhesion molecule 5 (CEACAM5) is a biomarker for various cancers, typically exhibiting minimal internalization. Goldenberg's team employed linkers containing acid-unstable carbonate or ester groups to conjugate SN-38 with the CEACAM5 antibody labetuzumab. The efficacy of these ADCs was assessed in mouse models with xenografts of colorectal cancer, pancreatic cancer, and systemic lymphoma. Encouragingly, despite the low internalization rate of CEACAM5, the ADCs induced substantial tumor growth inhibition, with those carrying the dual-cleavable linker CL2 demonstrating superior in vivo performance (Govindan et al. 2009). The authors proposed a noninternalizing mechanism in which SN-38 is gradually released extracellularly from the ADC, allowing for significant local accumulation of the free drug (Sharkey et al. 2011).

Govindan and colleagues on the CL2 and CL2A linker systems culminated in the clinical development and regulatory approval of sacituzumab govitecan, the first antibody–drug conjugate directed against Trop-2 (Cardillo et al. 2011). The compound's generic name, which incorporates elements of ‘Govindan’ and SN-38 – the active metabolite of irinotecan – reflects the foundational role of this chemistry in enabling clinical translation and highlights the impact of rational linker design in converting early-stage innovation into an approved therapeutic modality.

While current research trends may have shifted away from acid-labile linkers, these studies convincingly illustrate the feasibility of exploiting tumor microenvironment components for linker cleavage. These components may originate from tumor metabolism or be released upon tumor cell death.

#### 3.1.2. Disulfide linkers

Disulfide linkers are a prevalent category of cleavable linkers within ADCs, exemplified by Pfizer's Mylotarg's clinical success for the treatment of CD33-positive acute myeloid leukemia (Loke et al. 2014). These linkers are stable under physiological pH but can be susceptible to nucleophilic attack by serum albumin's (HSA) thiol groups, potentially leading to off-target effects (Bargh et al. 2019). Thomas H. Pillow innovated a linking technique that directly attaches small-molecule drugs to free thiols on a Cys-engineered antibody via disulfide bonds. This approach, exploiting protein steric hindrance, reduces the accessibility of the disulfide bond, thereby minimizing HSA interaction and enhancing linker stability in plasma (Pillow et al. 2017).

Glutathione (GSH) serves as an essential and abundant low-molecular-weight thiol antioxidant in cells, with concentrations in tumor cells approximately 1,000 times higher than in normal cells (Wang et al. 2021). Initial ADC designs leveraged this elevated cytosolic GSH to cleave linkers and release payloads intracellularly. However, subsequent findings revealed that disulfide linkers are also effective in noninternalizing ADCs, likely due to the release of high GSH levels by dying tumor cells into the tumor microenvironment, facilitating extracellular cleavage.

Polson et al. developed various ADCs containing linkers with noncleavable and cleavable disulfide bonds. *In vivo* results have shown that even against targets with minimal internalization, such as CD20, CD21, and CD72, cleavable disulfide-bond ADCs are still effective (Polson et al. 2009). Researchers hypothesized that disulfide linkers could be cleaved extracellularly, enabling noninternalizing mechanisms. These results challenge traditional views, highlighting that disulfide linkers can mediate payload release without internalization and confirming the possibility of extracellular linker cleavage for efficient ADC preparation.

Neri et al. presented a pioneering case of complete tumor regression in immunocompetent mice using a noninternalizing disulfide linker ADC. Fibronectin, a glycoprotein associated with angiogenesis, forms part of the extracellular matrix in the tumor vascular endothelium and is overexpressed in various human solid tumors, sparing most other tissues except the female reproductive system (Hooper et al. 2022). Researchers conjugated the toxic molecule DM1 to an F8 (SIP)-targeting fibronectin antibody using a disulfide linker, creating SIP(F8)-SS-DM1 (Bernardes et al. 2011). In F9 tumor-bearing models, SIP(F8)-SS-DM1 demonstrated robust antitumor activity and tumor selectivity, achieving complete tumor clearance. As tumor cells undergo apoptosis, they release increasing amounts of GSH into the microenvironment, accelerating ADC payload release and enhancing tumor cell death – a ‘self-amplification effect’.

In another study, Neri's team explored combining cytokines with ADCs to mitigate systemic cytokine side effects while enhancing efficacy. Using genetic engineering, they fused the immunostimulatory cytokine IL-2 with a bispecific F8 antibody and further linked it to the cytotoxic drug DM1 via a disulfide linker to construct a novel ADC (List et al. 2014). This ADC exhibited tumor targeting mediated by F8, immune activation by IL-2, and cytotoxicity by DM1. In F9 and C1498 tumor-bearing models, F8–IL2–SS–DM1 (0.5 mg/kg) induced a potent antitumor response, achieving complete tumor regression in some mice without significant adverse effects. While the molecular and cellular synergy between IL-2 and DM1 remains unidentified, the team hypothesizes that DM1-induced cytotoxic damage may enhance tumor immunogenicity and vascular permeability (Moschetta et al. 2012).

Galectin-3 binding protein (Gal-3-BP) is abundant and continuously secreted in most cancers, making it an ideal target for ADCs aimed at the cancer microenvironment. Stefano Iacobelli and colleagues developed a humanized antibody targeting Gal-3-BP's galectin-binding domain. Using disulfide bonds, they conjugated thiol-containing maytansinoid drugs (DM1, DM3, or DM4) at the C-terminal cysteine. The ADC employed microenvironmental reductants for disulfide bond cleavage, demonstrating significant tumor growth inhibition in vivo, with DM3 conjugates achieving long-term cures in mouse xenograft models (Giansanti et al. 2019).

The advent of immune checkpoint blockade (ICB) therapies has significantly reshaped cancer immunotherapy, with strategies such as blocking PD-1/PD-L1 interactions being among the most extensively studied (Wu et al. 2019; Lin et al. 2020). Other research indicates that toll-like receptor (TLR) agonists can activate TLR7/8 pathways, augmenting antitumor immune responses. However, systemic exposure to TLR agonists can induce proinflammatory cytokine release, causing severe adverse effects (Opal 2003).

To alleviate these side effects and propel clinical applications, Lei He's team proposed conjugating a PD-L1 antibody with a TLR agonist. They employed a self-immolative disulfide linker to couple D18 with the PD-L1 antibody avelumab, forming the ADC HE-S2. This ADC demonstrated good antitumor activity in mouse MC38 colon cancer and B16 melanoma models. This strategy not only reduced the required dose of the TLR agonist but also decreased its distribution to nontumor sites, significantly improving the dosing safety profile of the immune agonist (He et al. 2021).

#### 3.1.3. Enzyme-cleavable linkers

Lysosomes are abundant in proteolytic enzymes, presenting an opportunity for ADC linkers containing specific enzyme recognition sequences to facilitate payload release. Existing research has found overexpression of lysosomal enzymes extracellularly in various tumors. It has been confirmed that *β*-glucuronidase expression is significantly elevated in tumor tissues of breast, lung, ovarian, gastrointestinal cancers, as well as in melanoma (Wang et al. 2023). In recent years, prodrugs activated by *β*-glucuronidase in the tumor microenvironment have demonstrated promising clinical efficacy (Lu et al. 2025). These prodrugs feature a self-immolative linker connecting a glucuronide trigger to a cytotoxic drug, ensuring sufficient separation for the enzyme to easily recognize the glucuronide moiety. Enzymatic hydrolysis of the trigger subsequently activates the self-immolation reaction, leading to extracellular release of the active drug (Tranoy-Opalinski et al. 2014). The success of enzyme-activated tumor-targeting prodrugs offers new perspectives for ADC therapy. Researchers are now exploring whether lysosomal enzymes in the tumor microenvironment could cleave ADC linkers to release their active payloads. The widely used Val–Cit motif, originally optimized for cathepsin-B–dependent processing after internalization, may also be susceptible to extracellular proteases released during tumor cell death, a property that has enabled its opportunistic deployment in noninternalizing ADC formats and is further facilitated by the ready availability of commercial Val–Cit linker–payload modules (Conilh et al. 2023; Gorzen et al. 2025).

Neri's group pioneered investigations into utilizing tumor microenvironment enzymes to cleave linkers in noninternalizing ADCs. Building upon previous work with disulfide-cleavable linkers, they chose a monoclonal antibody targeting the noninternalizing antigen Tenascin-C splice variant, F16, for their studies (Schliemann et al. 2009). Tenascin-C is overexpressed in various tumors while exhibiting low expression in normal tissues (Dueck et al. 1999). Inspired by lysosomal enzyme-activated prodrug designs, researchers used a valine-citrulline (Val-Cit) dipeptide linker containing a self-immolative spacer to conjugate F16* with MMAE, creating the noninternalizing ADC IgG(F16)*-MMAE. This ADC exhibited superior plasma stability and achieved complete tumor remission in U87 and A431 xenograft models, whereas the SIP(F16)-MMAE group only showed significant tumor growth inhibition without complete cure (Gébleux et al. 2016).

To further enhance ADC blood stability and improve drug release efficiency, Neri's team investigated various dipeptide linker combinations, such as Val-Arg, Val-Ala, and Val-Lys, on IgG(F16)*-MMAE. Mass spectrometry revealed Val-Ala exhibited the lowest plasma degradation rate over 48 hours, producing the most potent therapeutic outcomes in A431 xenograft models. Notably, ADCs with noncleavable linkers like F16-NC-MMAE were ineffective, underscoring that the efficacy of noninternalizing ADCs critically depends on linker cleavage (Dal Corso et al. 2017).

Leucine-rich alpha-2-glycoprotein 1 (LRG1) is a secreted glycoprotein often induced under pathological conditions, promoting dysfunctional angiogenesis. Substantial evidence indicates that LRG1 is abundant in the microenvironment of many tumors and is closely related to tumor progression (Hong et al. 2019). Faiza Javaid's team selected an IgG4 monoclonal antibody, Magacizumab, targeting human LRG1, and, using a protease-sensitive Val-Cit linker and click chemistry, conjugated it to the cytotoxic drug MMAE. In vitro studies showed minimal internalization of this ADC, yet it induced significant cytotoxicity in LRG1-positive cells in the presence of exogenously supplied cathepsin B (IC50 = 0.9 nM). In vivo results demonstrated that the ADC effectively inhibited melanoma growth in mice, and combining it with low-dose cisplatin synergistically enhanced its efficacy. The authors hypothesize that cisplatin may induce cell death, releasing sufficient cathepsin B to activate the ADC, suggesting a combination strategy with other chemotherapeutics could improve the release efficiency of noninternalizing ADCs (Javaid et al. 2021).

Among extracellular proteases, fibroblast activation protein (FAP) has emerged as a particularly attractive target owing to its broad overexpression in solid tumors and restricted distribution in normal tissues (Nurmik et al. 2020) (Mona et al. 2022). Neri and colleagues pioneered FAP-activated conjugates using glycine–proline (Gly–Pro) dipeptide linkers that undergo rapid cleavage in the tumor microenvironment. Their OncoFAP–GlyPro–MMAE construct achieved high intratumoral payload concentrations while sparing healthy organs and produced durable antitumor activity in vivo (Backhaus et al. 2022). Subsequent comparative studies showed that FAP-cleavable conjugates outperformed analogous constructs bearing commercially derived linker–payload modules and supported further translational development (Zana et al. 2023; Bocci et al. 2024).

Beyond FAP, additional proteases have been harnessed for tumor-restricted release. Legumain, which is upregulated in multiple solid tumors and tumor-associated macrophages, has been exploited through engineered linker motifs that confer potent, tumor-selective ADC activation while limiting processing in healthy tissues (Khan et al. 2023). Matrix metalloproteinases (MMPs), which are frequently overexpressed during extracellular-matrix remodeling, have similarly been incorporated into modern designs, including modular bis-Fab constructs that remain stable in circulation but undergo tumor-local cleavage to release smaller, penetrant fragments with preserved antigen-dependent cytotoxicity (Gonzalez-Avila et al. 2019; Counsell et al. 2026).

Collectively, these studies establish extracellular protease-responsive linkers as a distinct and increasingly mature design paradigm for non- or poorly internalizing ADCs, expanding the therapeutic space beyond lysosomal processing and enabling new combination and targeting strategies.

Based on a bioinformatic screen of lysosomal enzymes, Christian Schröter's team identified *α*-l-iduronidase (IduA) as an ideal candidate for ADC linker cleavage due to its low expression in normal tissues and overexpression in various tumors. This study reported a novel IduA-cleavable ADC linker, employing exatecan and duocarmycin as payloads. They coupled iduronide-exatecan via interchain cysteines or iduronide-duocarmycin via microbial transglutaminase (mTG) to an anti-CEACAM5 (aCEA5) antibody. The iduronide-exatecan ADC not only demonstrated high serum stability but also demonstrated favorable tumor cell killing efficacy. Notably, it showed markedly reduced toxicity toward normal cells Compared to a cathepsin B-cleavable valine-citrulline-based exatecan ADCs. Furthermore, in vivo studies confirmed the significant antitumor efficacy of an IduA-cleavable duocarmycin ADC (Jäger et al. 2023).

### 3.2. Exogenously controlled cleavage

#### 3.2.1. Click-to-release and photo-sensitive linkers

Advances in chemical biology have introduced bioorthogonal reactions as powerful tools for selective ADC linker cleavage to release drug payloads (Fulton 2016; Yi et al. 2022). Unlike enzymatic and acid-induced cleavage, bioorthogonal reactions enable temporally and spatially controlled release without relying on cellular uptake or the tumor microenvironment. By externally introducing activators, precise release can be achieved, offering a promising new approach for ADC therapy (Fulton 2016). In 2016, Marc S. Robillard's team pioneered the use of bioorthogonal chemistry for ADC activation. They successfully achieved rapid dissociation and release of doxorubicin from a model ADC *in vitro* and in tumor-bearing mice using a newly developed inverse electron-demand Diels-Alder (IEDDA) pyridazine elimination reaction. They achieved rapid doxorubicin release in vitro and in tumor-bearing mice models, demonstrating the model ADC's high stability, tumor uptake, low off-target toxicity, high release efficiency, and effective tumor retention. This work laid the groundwork for future studies and marked the beginning of the ‘click-to-release’ era (Rossin et al. 2016).

Tumor-associated glycoprotein 72 (TAG-72) is a tumor-associated polymorphic epithelial mucin. It is highly expressed in human adenocarcinomas such as gastric, colon, breast, and lung cancers (Jelski and Mroczko 2020). Antibodies that bind TAG-72 tend to have limited shedding and do not internalize. The authors selected the anti-TAG72 monoclonal antibody mAb-CC49 and used the IEDDA pyridazine elimination reaction with TCO to conjugate mAb-CC49 to doxorubicin (Dox) as a model drug. Incorporation of a trans-cyclooctene linker into the ADC enabled facile ‘click-to-release’ chemistry with exogenously administered small-molecule tetrazine probes. To prevent premature drug release in the blood, they used an albumin-based scavenger that rapidly reacts with and clears TCO-labeled antibodies from the circulation, avoiding off-target toxicity.

In colon cancer xenograft mice, ADC uptake reached 30−40% ID/g in tumors 30 hours postinjection. The release rate was 51.3 ± 4.2% at 24 hours postactivator injection, which was comparable to the internalizing ADC release rates within tumors. Notably, very low levels of free doxorubicin were detected in the blood (0.7 ± 0.2%), demonstrating superior drug safety over current cleavable ADCs (Rossin et al. 2016).

To further enhance the clinical potential of click-release ADCs, Rossin's team developed a PEGylated CC49 diabody conjugate that clears from the blood faster than the first-generation mAb-CC49. The PEGylated CC49 diabody was efficiently taken up by tumors and completely cleared from the blood within two days. Diabodies have a smaller molecular weight than mAb-CC49 and lack an Fc region, thus improving penetration and potentially even reaching cancer stem cells deep within tumors. Animal studies indicated notable tumor growth inhibition using the activator 3 and 3 mg/kg tc-ADC, with median survival extended by 34 days. In multidose studies, repeated cycles of tc-ADC (3.75 mg/kg) combined with activator 3 led to significant and sustained tumor regression (CR = 7/8) (Rossin et al. 2018).

Recently, a myriad of new click-to-release reactions and photo-sensitive small molecules have emerged, offering expanded linker options for this ADC platform (Salma et al. 2018; Kobayashi et al. 2019; Zang et al. 2019; Sondag et al. 2022). The cleavage of some linkers can be triggered by ultraviolet or near-infrared light. These innovative release modalities mitigate challenges posed by tumor heterogeneity and patient variability, overcoming the limitations of insufficient or uncontrolled in vivo triggering.

#### 3.2.2. Radiation-active linker

Radiotherapy is a cornerstone of cancer treatment, employed in over 50% of cancer cases to eliminate malignant cells through ionizing radiation (Delaney et al. 2005). Modern techniques, such as three-dimensional conformal radiotherapy, enable precise tumor targeting, allowing for the delivery of high-dose radiation to tumors while minimizing damage to surrounding healthy tissues (Dearnaley et al. 1999). The clinical ubiquity, deep tissue penetration, and inherent targeting capability of radiotherapy render it an ideal exogenous stimulus for spatiotemporally controlled drug release strategies.

Although strategies utilizing radiotherapy to activate prodrugs were reported as early as the 1990s, their *in vivo* application has been hindered by issues of low efficiency and poor chemoselectivity (Tanabe et al. 2012). Within tumors, ionizing radiation primarily interacts with abundant water molecules rather than the relatively dilute prodrug molecules. From a radiation chemistry perspective, the mechanism by which these protecting groups capture hydrated electrons (e⁻_a_q) to form anion radical intermediates bears similarity to the single-electron transfer process in photoinduced electron transfer (PET) (Romero and Nicewicz 2016). PET-based cleavage reactions – where an electron transfers from a photoexcited sensitizer to a protecting group, leading to deprotection – have been extensively utilized for the controllable release of functional molecules.

Liu Lei and colleagues proposed that applying photoinduced electron transfer (PET) chemistry could open a new path for developing novel radiolytically removable protecting groups. They identified *N*-alkyl-4-methylpyridinium (NAP) as a highly efficient protecting group that rapidly releases payload molecules upon radiation, demonstrating cleavage efficiency superior to existing systems. Based on this, the team designed an NAP-derived carbamate linker that cleaves specifically during radiotherapy, releasing the toxin MMAE at the tumor site (Fu et al. 2024). This ADC showed significant efficacy in tumor-bearing mouse models, highlighting the great potential of this type of radiolytically removable protecting group for developing next-generation ADCs with higher stability and enhanced therapeutic potency.

Currently, several clinical trials and investigational new drug (IND) applications for noninternalizing ADCs are actively underway (Table 1). As of November 2025, multiple noninternalizing ADC candidates have advanced into clinical development, including PYX-201 (Pyxis Oncology), TGW101 (Tagworks), ABBV-085 (AbbVie), and YL242 (Yilian Biology).

**Table 1.** ADCs with cleavable linkers in clinical trials.

ADC type	ADC name	Target antigen	Linker	Cytotoxic payload	Clinical stage	Indications	Clinical trial number
Internalizing	M1231	MUC1/EGFR	vc	Hemiasterlin Derivative	Phase I	Solid tumors	NCT04695847
	BYON3521	cMET	vc	DUBA (Duocarmycin)	Phase I	Solid tumors	NCT05323045
	Cofetuzumab Pelidotin	PTK7	vc	Aur0101	Phase I	NSCLC	NCT03243331
	MRG001	CD20	vc	MMAE	Phase I	NHL	NCT06307522
	ORM-5029	HER2	vc	Smol007	Phase I	Solid tumors	NCT05511844
	SGN-LIV1A	LIV-1	vc	MMAE	Phase I	Breast cancer	NCT03310957
	CX-2029	CD71	vc	MMAE	Phase I/II	Solid tumors, DLBCL	NCT03543813
	MRG004A	TF	vc	MMAE	Phase I/II	Solid tumors	NCT04868162
	SYD985	HER2	vc	Duocarmycin	Phase I, II, III	Solid tumors, EC, BC	NCT04235101
	MRG003	EGFR	vc	MMAE	Phase I/II,	Solid tumors, NCBTC	NCT04868344
	EO-3021	CLDN18.2	Cleavable	MMAE	Phase I	Solid tumors	NCT05980416
	CS5001	ROR1	Cleavable	PBD	Phase I	Solid tumors and lymphomas	NCT05279300
	HS-20093	B7-H3	Cleavable	Topoisomerase I Inhibitor	Phase I/II	Solid tumors, ES-SCLC	NCT07186452
	AZD9592	EGFR/c-Met	Cleavable	Topoisomerase I Inhibitor	Phase I	Solid tumors	NCT06366451
	STRO-002	FRa	Cleavable	SC209 (Hemiasterlin)	Phase I	OC, EC	NCT03748186
	MGC018	B7-H3	Cleavable	Duocarmycin	Phase I/II	Solid tumors	NCT06227546
	Luveltamab Tazevibulin	FRa	Cleavable	3-Aminophenyl Hemiasterlin	Phase II	OC, FTC, PPC	NCT06679582
	MORAb-202	FRa	Disulfide Bond	Eribulin	Phase I/II	Solid tumors, NSCLC, OC, FTC, PPC	NCT04300556
	BAY94-9343	MSLN	SPDB	DM4	Phase I	Solid tumors	NCT03926143
Non-internalizing	TGW101	TAG-72	Click-to-release	MMAE	Phase I	Solid tumors	NCT06959706
	PYX-201	EDB of Fibronectin	mcValCitPABC-	Auristatin-0101	Phase I	Recurrent or refractory solid tumors	NCT05720117
	ABBV-085	LRRC15	-	MMAE	Phase I	Advanced solid tumors (e.g. sarcoma)	NCT02565758
	YL242	VEGF	TME-Activatable	-	Phase I	Solid tumors	NCT07197827

PYX-201 is a novel ADC targeting extradomain B (EDB) of fibronectin. It is currently being evaluated in a phase I clinical trial (NCT05720117) for safety, tolerability, and preliminary efficacy in patients with recurrent or refractory solid tumors, such as non-small cell lung cancer, breast cancer, and pancreatic ductal adenocarcinoma. Based on its promising therapeutic potential in pancreatic cancer, the U.S. Food and Drug Administration (FDA) has granted the PYX-201 Orphan Drug designation. In a phase I dose-escalation study, PYX-201 demonstrated an objective response rate (ORR) of 50% in patients with head and neck squamous cell carcinoma (HNSCC), including one complete response, along with a 100% disease control rate among six evaluable patients. Across the six solid tumor types included in the trial, an ORR of 26% was reported in 31 patients treated at therapeutically effective dose levels. These results support further clinical evaluation of PYX-201, both as a monotherapy and in combination regimens.

TGW101 represents a significant innovation through the application of bioorthogonal reversible chemistry, utilizing a ‘click-to-release’ mechanism. This ADC is administered intravenously and binds to the TAG-72 antigen expressed on the surface of tumor cells. Since TAG-72 is a noninternalizing target, conventional ADCs are unable to efficiently release their payload in this context. Following ADC localization, a small-molecule trigger is administered that selectively reacts with the ADC linker, inducing cleavage and release of the monomethyl auristatin E (MMAE) toxin within the extracellular tumor microenvironment, thereby exerting cytotoxic effects on neighboring tumor cells. According to ClinicalTrials.gov, a phase I study (NCT06959706) was initiated in May 2025. Recent updates indicate that the dose-escalation phase is progressing smoothly, having advanced to the third dose level by September 2025. Preliminary safety, pharmacokinetic, and early efficacy data are anticipated in early to mid-2026.

ABBV-085 is an ADC directed against LRRC15, a type I transmembrane protein expressed on mesenchymal-derived tumor cells (e.g. sarcomas) and commonly overexpressed on stromal fibroblasts across multiple solid tumors (e.g. breast, head and neck, and pancreatic cancers). As such, LRRC15 is considered a shared tumor and stromal antigen. ABBV-085 delivers MMAE to LRRC15-positive stromal cells; the released toxin, due to its cell-permeable nature, diffuses into the tumor microenvironment and mediates a potent bystander effect, killing adjacent proliferating tumor cells. A phase I clinical trial (NCT02565758) assessed ABBV-085 in patients with advanced solid tumors and found the drug to be generally well-tolerated. Promising antitumor activity was observed particularly in sarcoma patients, with an ORR of 20% in those with osteosarcoma and undifferentiated pleomorphic sarcoma, supporting the hypothesis that targeting LRRC15 may represent a viable therapeutic strategy for sarcomas (Demetri et al. 2021).

YL242 is an ADC targeting vascular endothelial growth factor (VEGF), which entered clinical trials (NCT07197827) in September 2025. Although VEGF is a well-validated oncology target, it is not amenable to traditional ADC strategies due to its extracellular localization. VEGF is highly enriched in peritumoral regions and plays a critical role in angiogenesis, with expression levels correlating with disease progression in multiple cancer types. The antibody component of YL242 has been extensively engineered, resulting in a 3- to 5-fold higher binding affinity for VEGF compared to conventional anti-VEGF antibodies. Preclinical studies in cynomolgus monkeys demonstrated a prolonged binding half-life of YL242 to VEGF of up to 72 hours, enabling sustained inhibition of VEGF signaling. YL242 incorporates Yilian Biology's next-generation tumor microenvironment-activatable linker-toxin platform, which confers high systemic stability, with a plasma half-life exceeding 120 hours. Upon entry into the acidic tumor microenvironment (pH ~6.5), the linker is rapidly cleaved, releasing the cytotoxic payload with an efficiency exceeding 85%. This environment-responsive design minimizes premature payload release in circulation, thereby enhancing tumor-specific targeting and reducing off-target toxicity.

As shown in Table 1, the majority of ADCs currently in clinical development target cell surface antigens that undergo antigen-mediated internalization, including HER2, EGFR, CD20, FRα, B7-H3, PTK7, LIV-1, and others. These ADCs primarily rely on protease-cleavable linkers (e.g. Val-Cit) and achieve payload release via endosomal/lysosomal processing (Gorzen et al. 2025). Of note, within the time frame of cytotoxicity assays, even slowly internalizing antigens may still support classical intracellular cleavage mechanisms, complicating the mechanistic attribution of payload release.

In contrast, Table 1 also highlights a distinct class of noninternalizing ADCs that target extracellular or matrix-associated antigens, including TAG-72 (TGW101) (Rossin et al. 2018), fibronectin EDB (PYX-201), LRRC15 (ABBV-085), and VEGF (YL242). These antigens are localized in the extracellular matrix or tumor microenvironment and are therefore intrinsically uncoupled from antigen-mediated endocytosis. Accordingly, such ADCs employ nonlysosomal release strategies – such as click-to-release chemistry or tumor microenvironment-activatable linkers – to ensure extracellular payload liberation that cannot be explained by delayed lysosomal trafficking.

## 4. Discussion

As an emerging field of ADCs, noninternalizing ADCs have made significant progress in the preclinical animal evaluation stage. However, current research on noninternalizing ADCs is still in its early phases, requiring the exploration of more reliable noninternalizing tumor-specific targets and novel linkers to ensure specific recognition and cleavage of noninternalizing ADCs. It is important to note that due to the absence of the tumor microenvironment in in vitro cell cultures, certain linkers dependent on endogenous metabolic products for cleavage may not be cleaved under these conditions. Consequently, results from in vitro experiments may not always align with in vivo outcomes for linkers sensitive to stimuli present in the tumor microenvironment.

As demonstrated by Zachary C. Hartman et al., the efficacy of trastuzumab deruxtecan (T-DXd) – a HER2-targeting antibody–drug conjugate – in HER2-low and HER2-negative breast cancers does not rely on HER2 binding or internalization of the ADC. Instead, it depends on extracellular proteases, such as cathepsin L, within the aggressive breast tumor microenvironment. This enzyme efficiently cleaves the cathepsin-specific linker in T-DXd, promoting payload release and thereby exerting cytotoxic effects on HER2-low/negative tumors (Tsao et al. 2025). This indicates that the extracellular cleavage and release mechanism is also crucial for targeting antigens with low expression or poor internalization efficiency.

Extracellular cleavage and release will significantly broaden the scope of targetable antigens for tumor therapy, including i) tumor stroma-associated antigens (e.g. Tenascin-C, Fibronectin extra domain-B (EDB), Collagen type IV (Lindgren et al. 2021), Periostin (POSTN) (Hong et al. 2013)), which are primarily localized in the extracellular matrix; ii) membrane proteins with specific glycosylation patterns or conformations (e.g. MUC1 (Pan et al. 2020), splice variants of PSMA (Bychkov et al. 2017; Jeitner et al. 2022), and CD44v6 (Ma et al. 2019)), whose structural features often lead to inefficient internalization; iii) specific subtypes or epitopes of immune checkpoint molecules (e.g. certain forms of PD-L1 (Xiao et al. 2021), VISTA (VSIR) (Chung et al. 2020), and CD47 (Chiang et al. 2022), which internalize slowly and are suitable for extracellular release strategies; iv) targets highly expressed in tumor vasculature or necrotic areas but with poor internalization (e.g. TEM8/ANTXR1 (Szot et al. 2018) and specific epitopes of Endoglin (CD105) (Huang et al. 2020) (Table 2).

**Table 2.** Potential targets for noninternalizing ADCs.

Target category	Target name	Localization	Internalization characterization
tumor stroma-associated antigens	Tenascin-C	Extracellular matrix	Non-internalization
	EDB	Extracellular matrix	Non-internalization
	Collagen type IV	Extracellular matrix	Non-internalization
	Periostin	Extracellularmatrix	Non-internalization
Membrane proteins with specific glycosylation patterns or conformations	MUC1	Tumor-associated membrane proteins	Poor-internalization
	PSMA	Tumor-associated membrane proteins	Poor-internalization
	CD44v6	Tumor-associated membrane proteins	Poor-internalization
Specific subtypes or epitopes of immune checkpoint molecules	PD-L1	Tumor-associated membrane proteins	Poor-internalization
	VISTA	Tumor-associated membrane proteins	Poor-internalization
	CD47	Tumor-associated membrane proteins	Poor-internalization
Targets highly expressed in tumor vasculature or necrotic areas	TEM8/ANTXR1	Tumor vasculature	Non -internalization
	CD105	Tumor vasculature	Non -internalization

Furthermore, considering tumor heterogeneity – where components like proteolytic enzymes vary significantly across cancer cell lines, cancer types, and species – a single linker strategy may not be universally applicable across all cancer types. This underscores the need to tailor linker strategies flexibly based on the specific antibody and cancer type involved. Given that the release of intracellular contents from dying tumor cells can further accelerate the cleavage of cleavable linkers, potentially resulting in a self-amplification effect, cautious exploration of appropriate dosing regimens in clinical settings is imperative. For patients in whom the microenvironment is less conducive to linker cleavage, combination therapies with chemotherapeutics and immunotherapy may be considered to enhance efficacy. In the future, with deepening understanding of the tumor microenvironment and continuous innovation in linker technology, particularly with the development of AI-assisted drug design, more potential extracellular ADC targets can be identified through high-throughput computational methods (Fauteux et al. 2015). Noninternalizing ADCs are expected to play an increasingly important role in the field of cancer therapy, providing patients with safer and more effective treatment options.

## Data Availability

Data sharing is not applicable to this article as no new data were created or analyzed in this research.
